# The Replication-Transmission Relativity Theory for Multiscale Modelling of Infectious Disease Systems

**DOI:** 10.1038/s41598-019-52820-3

**Published:** 2019-11-08

**Authors:** Winston Garira

**Affiliations:** Modelling Health and Environmental Linkages Research Group (MHELRG), Department of Mathematics and Applied Mathematics, University of Venda, Private Bag X5050, Thohoyandou, 0950 South Africa

**Keywords:** Nonlinear dynamics, Parasitic infection, Applied mathematics

## Abstract

It is our contention that for multiscale modelling of infectious disease systems to evolve and expand in scope, it needs to be founded on a theory. Such a theory would improve our ability to describe infectious disease systems in terms of their scales and levels of organization, and their inter-relationships. In this article we present a relativistic theory for multiscale modelling of infectious disease systems, that can be considered as an extension of the relativity principle in physics, called the replication-transmission relativity theory. This replication-transmission relativity theory states that at any level of organization of an infectious disease system there is no privileged/absolute scale which would determine, disease dynamics, only interactions between the microscale and macroscale. Such a relativistic theory provides a scientific basis for a systems level description of infectious disease systems using multiscale modelling methods. The central idea of this relativistic theory is that at every level of organization of an infectious disease system, the reciprocal influence between the microscale and the macroscale establishes a pathogen replication-transmission multiscale cycle. We distinguish two kinds of reciprocal influence between the microscale and the macroscale based on systematic differences in their conditions of relevancy. Evidence for the validity of the replication-transmission relativity theory is presented using a multiscale model of hookworm infection that is developed at host level when the relationship between the microscale and the macroscale is described by one of the forms of reciprocal influence.

## Introduction

For more than a century now, the study of infectious disease systems has been informed by two main theories. These two theories are the infectious disease causation theory which is a series of theories which were progressively refined (as new knowledge emerged) to explain the causes of infectious diseases and the infectious disease transmission mechanism theory which sought to use knowledge of infectious disease transmission mechanisms to develop quantitative models of infectious disease dynamics. In what follows we briefly describe each of these two theories.

- *The infectious disease causation theory*: This is not a single theory, but a series of theories which were progressively refined, one after another, to explain the cause of infectious disease systems. The successive infectious disease causation theories were challenged and changed as new knowledge on infectious disease causation was developed, changing people’s views about the causes of infectious diseases. The initial infectious disease causation theory changed from before the rise of modern medicine, when infectious diseases were attributed to a variety of spiritual and demonic forces including punishment from God for sinful behaviour or weak moral character or as a result of witchcraft to the era of modern medicine. In the era of modern medicine, infectious disease causation theory changed from being based on super natural forces (demonic theory and punitive theory) to being looked upon as caused by natural processes. This involved moving medicine from magic to give it a scientific basis. Within this era, infectious disease causation theory moved from a miasmic theory which was based on the inference that the air arising from certain kinds of ground, especially low swampy areas was the cause of infectious disease, to the germ theory which postulate that infectious diseases are caused by germs/microbes/pathogens up to the current epidemiological triad theory which states that an infectious disease system is a result of the interaction of three sub-systems which are: (i) the host sub-system, (ii) the pathogen sub-system, and (iii) the environment sub-system resulting in infectious disease systems being organized into hierarchical multilevel and multiscale complex systems with levels ranging from the cellular level to the macroecosystem level. Therefore, the epidemiological triad theory constitutes the current and modern infectious disease causation theory. For more details on the changing forms of infectious disease causation theory see^1^.
- *The infectious disease transmission mechanism theory*: This theory is based on the idea that infectious disease dynamics consist of transmission as the main dynamic disease process at each hierarchical level (the cell level, the tissue level, the host level, etc.) and that specific transmission models can be developed to study an infectious disease system at a particular level of organization. The standard approach in the development of such transmission models at any level is to classify the population (which may be a population of cells for the cell level or a population of tissues such as granulomas for the tissue level, or a population of hosts for the host level) into compartments within which individuals (cells, tissues, hosts, etc.) behave homogeneously. These models have been used to aid understanding of infectious disease transmission dynamics and increase our capabilities for control of infectious diseases with fewer resources. Within this theory infectious disease dynamics is thought to be a result of three main transmission mechanisms which are as follows. (i) *Direct transmission mechanism*: in which transmission models are developed based on compartmentalizing the population (cells, tissues, hosts, etc.) into susceptible, exposed, infected, recovered (SEIR), and variations of this paradigm (SI, SIS, SEI, SEIS, SIR, SIRS, SEIRS, etc.) at each hierarchical level (the cell level, the tissue level, the host level, etc.) of an infectious disease system. (ii) *Environmental transmission mechanism*: in which transmission models are developed based on compartmentalizing the population (cells, tissues, hosts, etc.) into susceptible, exposed, infected, recovered, and environmental pathogen load (SEIRP), and variations of this paradigm (SIP, SISP, SEIP, SEISP, SIRP, SIRSP, SEIRSP, etc.). The obvious distinction between transmission models for directly transmitted infectious disease systems and environmentally transmitted infectious disease systems is that the latter usually have at least one extra equation describing the dynamics of pathogen in the environment. (iii) *Vector-borne transmission mechanism*: which arise because the pathogen has a complex life cycle so that there is need for two hosts (a vertebrate host and vector host) for the pathogen to complete its life cycle. These infectious disease systems can be environmentally transmitted such as in schistosomiasis where transmission models at host level are developed by compartmentalizing the host population into susceptible, exposed, infected, recovered, and environmental pathogen load (SEIRP), and variations of this paradigm (SIP, SISP, SEIP, SEISP, SIRP, SIRSP, SEIRSP, etc.) or directly transmitted such as malaria where transmission models at host level are developed by compartmentalizing the host population into susceptible, exposed, infected, recovered (SEIR), and variations of this paradigm (SI, SIS, SEI, SEIS, SIR, SIRS, SEIRS, etc.) in the context of two-host infections (the vertebrate host and the vector host).

However, while the current infectious disease causation theory in the form of the epidemiological triad theory is adequate to explain the causes of infectious diseases, the infectious disease transmission mechanism theory, which considers infectious disease transmission as the main dynamic disease process, is not capable of providing a systems level description of infectious disease systems using multiscale modelling approaches. Therefore, the infectious disease transmission mechanism theory is not adequate to describe infectious disease phenomena that vary through time and space and at different scales. This is expected because transmission at any level of organization of an infectious disease system (the cell level, the tissue level, the host level, etc.) is a single scale disease process. In order to describe infectious disease phenomena that vary through time and space and at different scales, we propose a new theory which incorporates events (that is, pathogen replication) that give rise to transmission and thus accommodate variation in time and space. The proposed new theory for multiscale modelling of infectious disease systems, which incorporates pathogen replication is called the replication-transmission relativity theory. This relativity theory states that at any level of organization of an infectious disease system there is no privileged/absolute scale which would determine, disease dynamics, only interactions between the microscale and macroscale. It acknowledges that at every level of organization of an infectious disease system, pathogens must succeed at both the microscale (where pathogen replication often occurs) and the macroscale (where pathogen transmission often occurs) if they are going to spread and persist at a particular level of organization of an infectious disease system. This replication-transmission relativity theory is a powerful supporting conceptual tool to develop a systems level description of infectious disease systems using multiscale modelling methods. It is an extension of the relativity principle in physics. In physics, the relativity principle makes a powerful and specific statement that there is no absolute/privileged frame of reference from which to determine motion of objects and other physical quantities such as position/location, velocity, and acceleration (see^2^ for more details). The replication transmission relativity theory is an extension of the relativity principle in physics by requiring that at every level of organization of an infectious disease system, the scales of observation (i.e. the microscale and the macroscale) be considered as characterizing frames of reference.

Our idea of the relativity theory for multiscale modelling of infectious disease systems makes distinction between a level and a scale. It assumes that within a level there are two limiting adjacent scales of infection^3,4^ which are a microscale and a macroscale. Although it is a feature of complex systems that they are multilevel and multiscale, these complex systems are organized differently in that for some complex systems a scale is the same as a level, while for other complex systems, a level is different from a scale. Infectious disease systems are one example of complex systems in which a scale is different from a level. However, for some complex systems such as the immune response system at the site of infection, a level is the same as a scale. Therefore, for the immune response system at the site of infection, we can interchangeably use the words level and scale when we, for example, state that the immune response system at the site of infection is organized into three main levels/scales which are (i) the molecular level/scale, (ii) the cellular level/scale, and (iii) the tissue level/scale. For complex systems such as infectious disease systems in which a level is different from a scale, each level can be resolved into two limiting adjacent scales^3,4^ which are a microscale and a macroscale. This implies that the dynamics of an infectious disease system at a particular level of organization is a multiscale loop involving a microscale and a macroscale. Therefore, consideration of the multiscale dynamics of an infectious disease system at any of its levels of organization requires consideration of the dynamics at two adjacent scales: a microscale and a macroscale.

## The Hierarchical Levels of Organization of an Infectious Disease System

**Figure 1:**
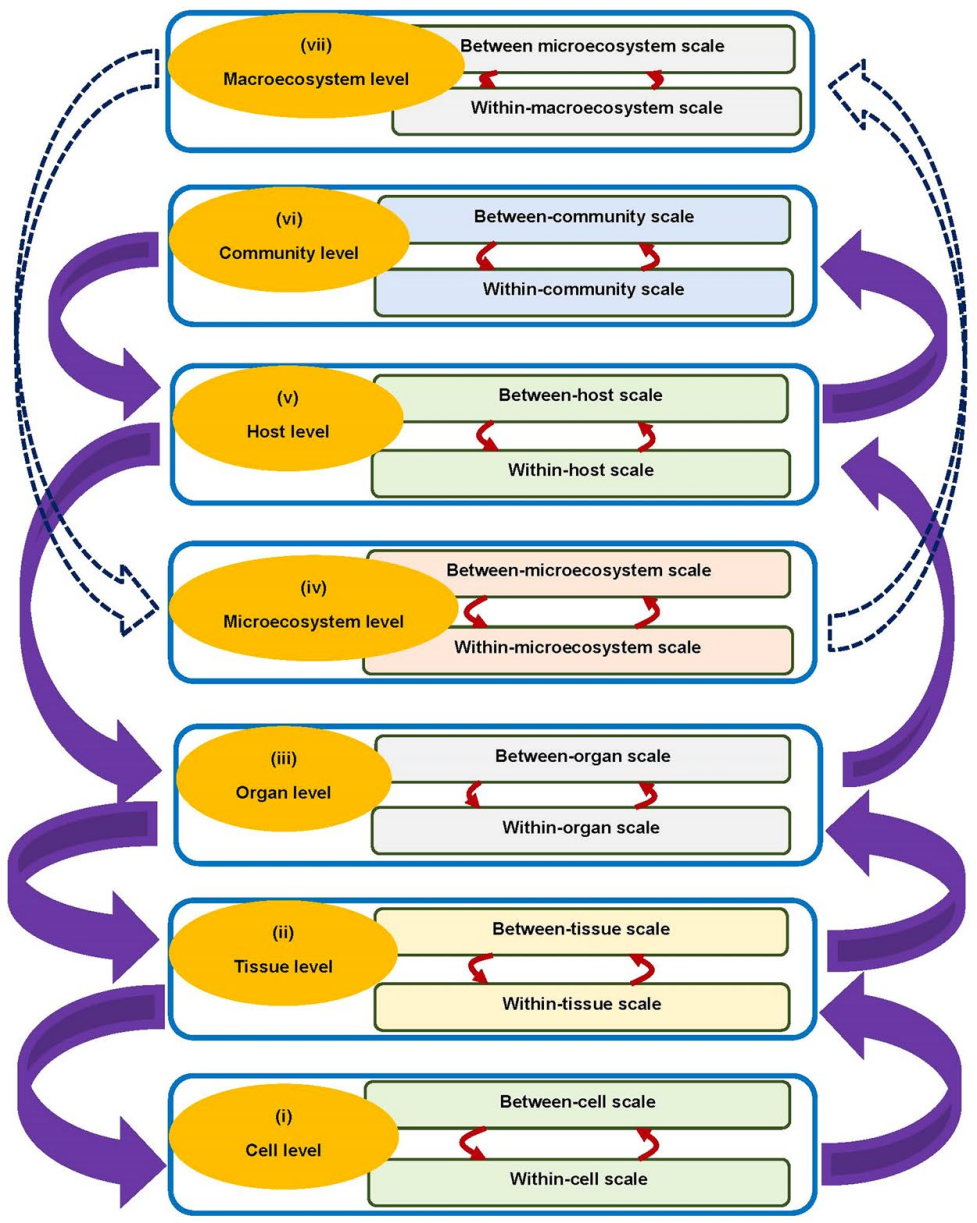
Conceptual diagram of the seven hierarchical levels of organization of an infectious disease system and the associated macroscale and microscale for each hierarchical level.

Although the hierarchical, multilevel and multiscale nature of infectious disease systems has been discussed in detail in our previous works^3,4^, here we build on this foundational work and propose that infectious disease systems are organized into seven main hierarchical levels. These seven levels of organization of an infectious disease system are conceptually represented in Fig. 1. From Fig. 1, we notice that each of these levels of organization of an infectious disease system has two limiting adjacent scales, that is, a microscale and a macroscale. Thus within each of the seven main levels of organization of an infectious disease system, there exists some open scale boundary (which allows for bidirectional flow of information between scales) which divides a level into two adjacent scales (a microscale and a macroscale), making infectious disease dynamics at each hierarchical level a multiscale loop involving the reciprocal (i.e. both way) influence of the macroscale and microscale. We briefly describe each of the seven levels of organization of an infectious disease system in Fig. 1 and the associated two limiting adjacent scales (a microscale and a macroscale) as follows. (i) *The cell level*: This level of organization has the within-cell scale and between-cell scale as its microscale and macroscale respectively. Different types of target cells can be considered in the multiscale dynamics of infectious disease dynamics such as *CD*4^+^ T cells (for HIV) or red blood cells (for malaria) when integrating the within-cell scale and between-cell scale. (ii) *The tissue level*: This level of organization has the within-tissue scale and between-tissue scale as its microscale and macroscale respectively. The different types of tissues that can be considered in the multiscale dynamics of infectious disease systems include granuloma^5^ for tuberculosis or microabscess^6^ caused by some bacterial infections. (iii) *The organ*/*anatomical compartment level*: The microscale and macroscale for this level of organization of an infectious disease system are the within-organ/anatomical compartment scale and the between-organ/anatomical compartment scale. Infectious disease systems at this level are described in terms of single pathogen species/strain and multiple organs/anatomical compartments. Some of the organs/anatomical compartments considered at this level of organization of an infectious disease system are the lung, brain, gut, kidney, muscle, heart, pancreas, stomach, liver, spleen, bone, adrenal, skin, adipose, and blood. (iv) *The microecosystem level*: At this level of organization of an infectious disease system, the different organs/anatomical compartments (lung, gut, kidney, heart, stomach, liver, skin, blood, etc.) are considered as ecosystems. Therefore, infectious disease systems at this level are described in terms of multiple organs/anatomical compartments and multiple pathogen species/strains. The microscale and macroscale for this level of organization of an infectious disease system are the within-microecosystem scale and the between-microecosystem scale respectively. Because of the multiple pathogen species/strains interactions at this level, ecological process/interactions influence infectious disease dynamics which include the competitive species/strains interactions and the mutualistic interactions between the multiple pathogen species/strains. (v) *The host*/*organism level*: This level of organization has the within-host scale and between-host scale as its microscale and macroscale respectively. Infectious disease systems at this level are described in terms of single pathogen species/strain as well as single host species and single community. We will use this level to illustrate the validity of the replication-transmission relativity theory when the form of reciprocal influence between the within-host scale (microscale) and the between-host scale (macroscale) consists of both super-infection, that is, repeated infection before the host recovers from an infectious episode (for the influence of between-host scale on within-host scale) and pathogen excretion/shedding (for the influence of within-host scale on between-host scale) using a multiscale model for hookworm disease system. (vi) *The community level*: The microscale and macroscale for this level of organization of an infectious disease system are the within-community scale and the between-community scale. Infectious disease systems at this level are described in terms of single pathogen species/strain as well as single host species and multiple communities. Some of the communities considered at this level of organization of an infectious disease system are local community (village, district, town, province, etc.), territorial community (i.e. nation) and regional community (e.g. the six World Health Organization (WHO) regions of the world: African Region, Region of the Americas, South-East Asia Region, European Region, Eastern Mediterranean Region, and Western Pacific Region). (vii) *The macroecosystem level*: At this level of organization of an infectious disease system, the different communities (local, national, regional, etc.) are considered as ecosystems. Therefore, infectious disease systems at this level are described in terms of multiple communities and/or multiple pathogen species/strains. The microscale and macroscale for this level of organization of an infectious disease system are the within-macroecosystem scale and the between-macroecosystem scale respectively. Because of the multiple species interactions at this level of organization of an infectious disease system, ecological process/interactions influence infectious disease dynamics which include predator/prey interactions, competitive pathogen and/or host species interactions and the mutualistic interactions between the multiple pathogen species/strains and/or multiple host species. Although the interaction of levels is also shown in Fig. 1 (shown in wide purple arrows and wide white arrows with dotted lines), discussion on the interactions between levels is beyond the scope of this article. Notable among the interactions between levels are the interactions between the microecosystem level and the macroecosystem level (shown in wide white arrows with dotted lines) which are relevant for multiscale modelling of the ecology and evolution of infectious disease systems.

## The Reciprocal Influence of Macroscale and Microscale at Hierarchical Levels of an Infectious Disease System

**Figure 2:**
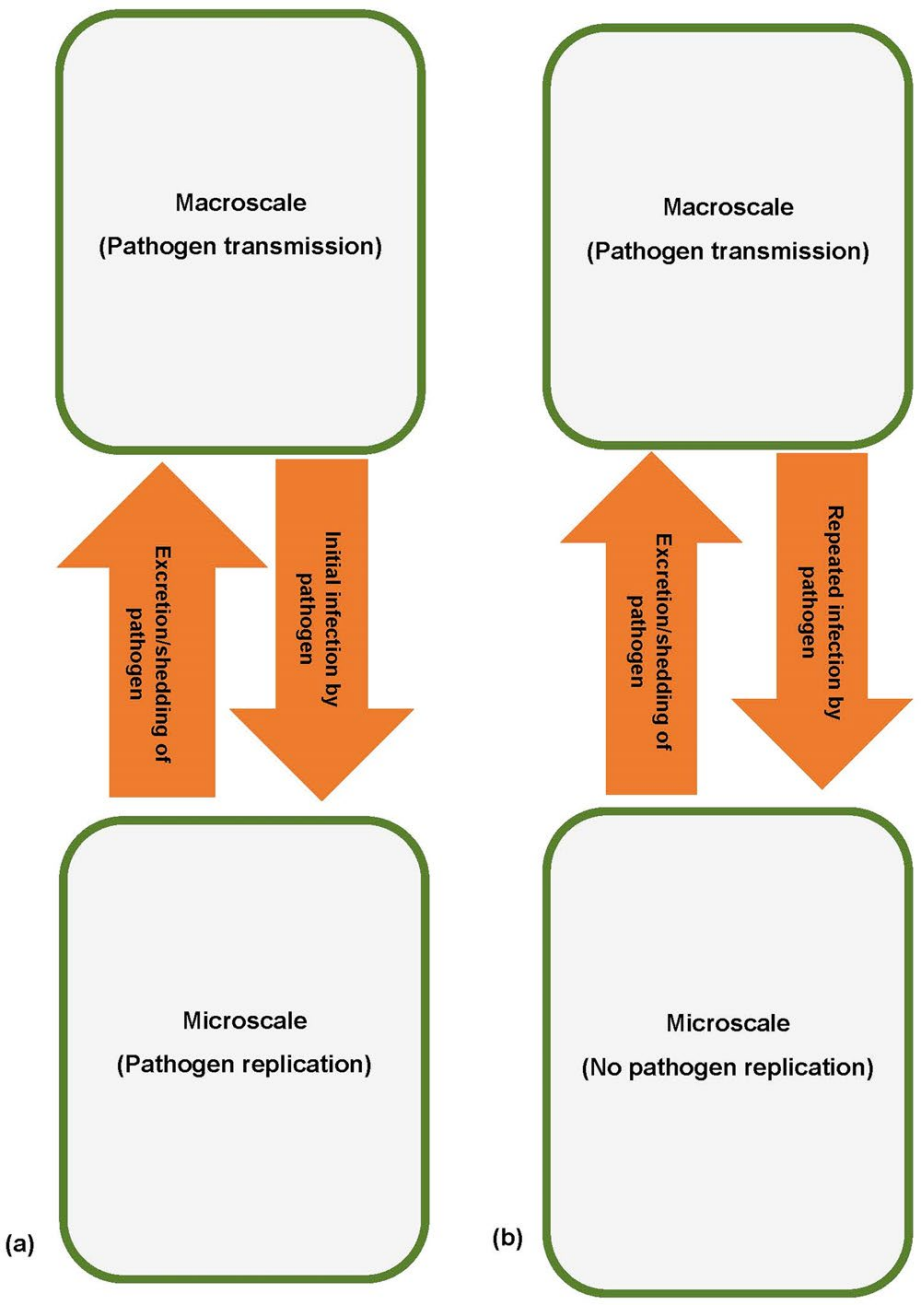
A conceptual diagram of the two different types of reciprocal influence between the macroscale and microscale at hierarchical levels of organization of infectious disease systems. (**a**) Type I reciprocal influence between the macroscale and microscale within a hierarchical level and (**b**) type II reciprocal influence between the macroscale and microscale within a hierarchical level.

The replication-transmission relativity theory for multiscale modelling of infectious disease systems presented in this article implies that infectious disease dynamics is faced with circular causality of disease processes at each of the seven hierarchical levels of organization. This is because at each of these hierarchical levels of organization, the microscale and the macroscale influence each other in a reciprocal manner. The fundamental mechanism of how the microscale and the macroscale influence each other in a reciprocal way at each of the hierarchical levels of organization of an infectious disease system is through interaction of four key disease processes which are: (i) infection/super-infection by pathogen, (ii) pathogen replication, (iii) pathogen shedding/excretion and (iv) pathogen transmission. At each of the hierarchical levels of organization of an infectious disease system, disease dynamics involves a pathogen replication-transmission multiscale cycle. This replication-transmission multiscale cycle happens because at each of the hierarchical levels of organization of an infectious disease system, the characteristic scale at which pathogen replication and pathogen transmission occur often do not match. At each level the macroscale influences the microscale through infection/super-infection (which involves movement of pathogen from macroscale into the microscale) while the microscale influences the macroscale through pathogen shedding/excretion (which also involves movement of pathogen from microscale into the macroscale). Therefore, at each of the hierarchical levels of organization of an infectious disease system, infection/super-infection and shedding/excretion of the pathogen introduce a multiscale cycle of influence between pathogen replication at the microscale and pathogen transmission at the macroscale. For this reason, disease dynamics at the microscale alone or macroscale alone at each hierarchical level restricts itself to one part of the replication-transmission multiscale cycle. For the full understanding of disease dynamics at each of the hierarchical levels of an infectious disease system, one has to close the replication-transmission multiscale loop and consider the full feedback between the microscale pathogen replication processes and the macroscale transmission processes. In the context of the replication-transmission relativity theory, we propose that there are two different types of the reciprocal influence between the microscale and the microscale (type I reciprocal influence and type II reciprocal influence). Figure 2 shows a conceptual representation of these two types of reciprocal influences between the microscale and the macroscale. The nature of these reciprocal influences are summarized as follows:

- *Type I reciprocal influence between the macroscale and microscale within a hierarchical level*: In this type of reciprocal influence, the microscale influences the macroscale through pathogen shedding/excretion. This involves the movement of pathogen from the microscale to the macroscale. Further, the macroscale influences the microscale through initial infection of the microscale which involves movement of pathogen from macroscale into the microscale. From a mathematical point of view the macroscale in this case influences the microscale through initial conditions of the microscale submodel variables (initial infection).
- *Type II reciprocal influence between the macroscale and microscale within a hierarchical level*: In this type of reciprocal influence, the microscale also influences the macroscale through pathogen shedding/excretion. As in type I reciprocal influence, this involves the movement of pathogen from the microscale to the macroscale. However, the macroscale influences the microscale through super-infection (i.e. repeated infection before the host recovers from an infectious episode) which also involves movement of pathogen from the macroscale to the microscale. From a mathematical point of view the macroscale influences the microscale through scaled down macroscale variables and parameters.

Using these two types of reciprocal (i.e., both way) influence between the microscale and the macroscale at hierarchical levels of organization of an infectious disease system which are part of the replication-transmission relativity theory, we can make more precise statements about the five different categories of multiscale models of infectious disease systems given in^3,4^. At the time of categorization of multiscale models of infectious disease systems in^3,4^, we did not have knowledge of the existence of type I reciprocal influence which is now part of the dictates of the replication-transmission relativity theory. As a result, nested multiscale models and some classes of hybrid multiscale models were considered to be those multiscale models where there is no influence of the macroscale on the microscale. However, in the light of replication-transmission relativity theory which dictates that there is always reciprocal influence between the microscale and the macroscale, we make the following precise statements about the five categories of multiscale models of infectious disease systems in^3,4^.

1. *Category I - Individual Based Multiscale Models* (*IMSMs*). A generic category of multiscale models that provides a simplified means of describing infectious disease dynamics at a particular level of organization of an infectious disease system while incorporating individual heterogeneity in which the microscale submodel and the macroscale submodel are integrated through type I reciprocal influence. In this category of multiscale models, the macroscale is often observed as emergent behaviour of the microscale entities.
2. *Category II - Nested Multiscale Models* (*NMSMs*). A generic category of multiscale models that provides a simplified means of describing infectious disease dynamics at a particular level of organization of an infectious disease system in which the microscale submodel and the macroscale submodel are integrated through type I reciprocal influence. The microscale submodel and the macroscale submodel must be described by the same formalism or mathematical representation.
3. *Category III - Embedded Multiscale Models* (*EMSMs*): A generic category of multiscale models that provides a simplified means of describing infectious disease dynamics at a particular level of organization of an infectious disease system in which the microscale submodel and the macroscale submodel are integrated through type II reciprocal influence. The microscale submodel and the macroscale submodel must be described by the same formalism or mathematical representation.
4. *Category IV: Hybrid multiscale models* (*HMSMs*): A generic category of multiscale models that provides a simplified means of describing infectious disease dynamics at a particular level of organization of an infectious disease system in which the microscale submodel and the macroscale submodel are integrated through either type I reciprocal influence or through type II reciprocal influence. However, in this case the microscale submodel and macroscale submodel are described by different mathematical representations. Examples of such paired formalisms are deterministic/stochastic, discrete time/continuous time, mechanistic/phenomenological, ODE/PDE, ODE/ABM, ODE/CA, etc.
5. *Category V - Coupled multiscale Models* (*CMSMs*): A generic category of multiscale models that provides a simplified means of describing infectious disease dynamics at multiple levels of organization of an infectious disease system by either integrating a single scale submodel and some multiscale models from any of categories I, II, III, and IV (for multiscale models that integrate more than two scales which are odd in number) or by integrating multiscale models from any of categories I, II, III or IV (for multiscale models that integrate more than two scales which are even in number) either through type I reciprocal influence or through type II reciprocal influence or a combination of both. The multiscale models developed in this category consider multiple pathogen strain infections, and/or multiple pathogen species infections, and/or multiple host group infections, and/or multiple host species infections, and/or multiple communities infections, and/or multiple organ/anatomical compartment infections. They are not like categories I, II, III, and IV which focus on a specific combination of (i) one-host and (ii) one-pathogen species/strain and (iii) one-level of organization of an infectious disease system relationships in multiscale modelling. Therefore, reciprocal influence between levels is characterized by coupled multiscale models.

These five categories of multiscale models of infectious disease systems are still the same as in^3,4^, except that where the categorization in^3,4^ indicates that there is no influence of the macroscale on the microscale, we now in line with the specifications of the replication-transmission relativity theory explicitly state that there is type I reciprocal influence between the macroscale and the microscale. For a detailed review of the multiscale models in the five different categories that have been developed at each hierarchical level of an infectious disease system see^3^. In this article, we use a multiscale model of hookworm infection, an environmentally transmitted infectious disease system to illustrate the validity of the replication-transmission theory when the relationship between the microscale and the macroscale is described by type II reciprocal influence at host level of the infectious disease system. In general, environmentally transmitted infectious disease systems are infectious disease systems in which the infectious agent (the pathogen) has some free-living life stages in the geographical environment’s physical entities (soil, water, contact surfaces, air, food, etc.). When the dynamics of such infectious disease systems are considered at host level (which has the within-host scale and the between-host scale as its microscale and macroscale respectively), the transmission of the disease at between-host scale is facilitated by a free-living life stage of the pathogen in the geographical environment’s physical entities (soil, water, contact surfaces, air, food, etc.). We identify three different types of environmentally transmitted infectious disease systems which are:

- *Type I environmentally transmitted infectious disease systems*: These are environmentally transmitted infectious disease systems in which there is no pathogen replication at the microscale (within-host scale) such as schistosomiasis^7^, Guinea worm^8^, and soil transmitted helminths infections such as hookworm^9^. This is particularly true for helminths infections because, with few exceptions (Strongyloides, Trichinella, some tapeworm larvae), helminths do not increase their numbers within a host by replication at the microscale (within-host scale). For these infectious disease systems, the pathogen load at the within-host scale is directly related to the number of infective stages encountered by the host (i.e. taken up by the host) in the environment (water, soil, food, air, contact surfaces, etc.) through super-infection (i.e. repeated infection before the host recovers from an infectious episode). For such environmentally transmitted infectious disease systems host behaviour is a major factor influencing the disease burden in a particular community because certain ways of behaving, particularly with regard to sanitation and hygiene will result in greater disease transmission. Such infectious disease systems can be modelled by embedded multiscale models in which type II reciprocal influence describes the relationship between the microscale and the macroscale at host level of organization of an infectious disease system.
- *Type II environmentally transmitted infectious disease systems*: These are environmentally transmitted infectious disease systems in which the pathogen only replicates at the microscale (within-host scale). Some viral infections such as influenza^10^ and some bacterial infections such as paratuberculosis species^11^, and Mycobacterium tuberculosis^12^ are good examples of Type II environmentally transmitted infectious disease systems. Such infectious disease systems can be modelled by nested multiscale models in which type I reciprocal influence describes the relationship between the microscale and the macroscale at host level of organization of an infectious disease system.
- *Type III environmentally transmitted infectious disease systems*: These are environmentally transmitted infectious disease systems in which the pathogen replicates at both the microscale (within-host scale) and at the macroscale (between-host scale). Such environmentally transmitted infectious disease systems are caused by opportunistic infections such as cholera^13^, Salmonella enterica^14^ and anthrax^15^. For these infectious disease systems a combination of type I reciprocal influence and type II reciprocal influence describe the relationship between the microscale and the macroscale at host level of organization of an infectious disease system.

In what follows we develop the multiscale model for hookworm infection at host level which is later used to illustrate the validity of the replication-transmission relativity theory when the relationship between the microscale and the macroscale is described by type II reciprocal influence. As noted earlier, type II reciprocal influence consists of (i) super-infection (i.e. repeated infection before the host recovers from the infectious episode) - for the influence of between-host scale on within-host scale and (ii) pathogen shedding/excretion – for the influence of within-host scale on between-host scale. However, we begin the multiscale modelling by developing a general multiscale model for an environmentally transmitted infectious disease system of type I at host level. This general multiscale model is then applied to hookworm infection as a paradigm. We start by developing a general multiscale model so that those who want demonstrate the validity of the replication-transmission relativity theory at host level of organization of an infectious disease system by developing their own multiscale model for a different environmentally transmitted infectious disease system of type I know where to start.

## The General Multiscale Model for Environmentally Transmitted Infectious Disease Systems of Type I

While the replication-transmission relativity theory states that at any of the seven main hierarchical levels of organization of an infectious disease system there is always reciprocal influence between the microscale and the macroscale, the tools we have to establish this nature of such cross scale influences between the microscale and the macroscale need to be further developed for different infectious disease systems with different transmission pathways that include (i) direct transmission, (ii) environmental transmission, and (iii) vector-borne transmission. Consider a general environmentally transmitted infectious disease system of type I in which hosts are compartmentalized into two compartments according to their disease status, that is, susceptible hosts *S*_*H*_ and infected hosts *I*_*H*_, with no natural recovery of infected hosts. Suppose that *Y*_0_ = *P*_*F*_ is the pathogen population in the first life stage in the environment and *Y*_*n*_ = *P*_*H*_ is the pathogen population in the last life stage in the environment (also assumed to be the infectious life stage) while *Y*_1_, *Y*_2_, ……, *Y*_*n*−1_ are the pathogen populations in the other intermediate life stages in the environment (i.e. at the macroscale). Further, suppose that *X*_0_ = *P*_*f*_ is also the pathogen population in the first life stage in the infected host and *X*_*m*_ = *P*_*h*_ is the pathogen population in the last life stage in the infected host (also assumed to be the life stage that is excreted/shed into the environment) while *X*_1_, *X*_2_, ……, *X*_*m*−1_ are the pathogen populations in the other intermediate life stages in the infected host (i.e. at the microscale). We now develop a general multiscale model of an environmentally transmitted infectious disease system of type I at host level that integrates the two sets of variables which are (i) *S*_*H*_, *I*_*H*_, *Y*_0_, *Y*_1_, …, *Y*_*n*_ at between-host scale (the macroscale), and (ii) *X*_0_, *X*_1_, …, *X*_*m*_ at within-host scale (the microscale).

If we assume the presence of a type I environmentally transmitted infectious disease with only one host species involved in the transmission of the environmentally transmitted infectious disease system, then this results in a multiscale model involving human-to-environment and environment-to-human transmission components at host level. The fundamental theory and science for development of such environmentally transmitted infectious disease systems of type I at host level was presented in our previous paper^7^ in the context of an environmentally transmitted vector-borne disease system, and in particular, human schistosomiasis. Here we make some refinements that establishes this multiscale modelling approach in a more general way. The two central lessons to emerge from^7^, about multiscale modelling of the reciprocal influence between the microscale and the macroscale for environmentally infectious disease systems of type I at host-level are that this reciprocal influence can be modelled in the following way.

(i) The influence of the between-host scale (the macroscale for the host level) on the within-host scale (the microscale for the host level) through super-infection can be modelled by down-scaling between-host scale uptake of the pathogen in the environment through infection of hosts at the population level at a rate *β*_*H*_*λ*_*H*_(*P*_*H*_)*S*_*H*_ to repeated infection of the individual host at a rate $\frac{{\beta }_{H}{\lambda }_{H}({P}_{H})[{S}_{H}(t)-1]}{{\Phi }_{H}[{I}_{H}(t)+1]}$. However, this representation of super-infection is a refinement of the approach in^7^ in two ways. First, the approach in^7^ specifies the functional form of *λ*_*H*_(*P*_*H*_). Here *λ*_*H*_(*P*_*H*_) is a general class of functions whose properties will be specified shortly. Secondly, the approach in^7^ over-estimated the number of new infections, while here the number of new infections is assumed to be a proportion Φ_*H*_ of the existing cumulative number of infections.
(ii) The influence of the within-host scale (the microscale for the host level) on the between-host scale (the macroscale for the host level) through pathogen shedding/excretion can be modelled by up-scaling individual host excretion/shedding of the pathogen from within-host scale at a rate *α*_*h*_*P*_*h*_ to between-host scale. This up-scaling of individual excretion/shedding of pathogen is modelled by [*I*_*H*_(*t*) + 1]*α*_*h*_*P*_*h*_(*t*).

Based on these fundamental ideas, which represent a refinement of the work in^7^, then the casual links between the state variables at the microscale and macroscale described above can be recast into a general multiscale model of an environmentally transmitted infectious disease system of type I at host level in the form:

1 $$\begin{array}{ccc}1.\,\frac{d{S}_{H}(t)}{dt} & = & {\Lambda }_{H}-{\beta }_{H}{\lambda }_{H}({P}_{H}){S}_{H}(t)-{\mu }_{H}{S}_{H}(t),\\ 2.\,\frac{d{I}_{H}(t)}{dt} & = & {\beta }_{H}\lambda ({P}_{H}){S}_{H}(t)-({\mu }_{H}+{\delta }_{H}){I}_{H}(t),\\ 3.\,\frac{d{P}_{F}(t)}{dt} & = & [{I}_{H}(t)+1]{\alpha }_{h}{P}_{h}(t)-({\nu }_{F}+{\gamma }_{F}){P}_{F}(t),\\ 4.\,\frac{d{Y}_{i}(t)}{dt} & = & {F}_{i}({Y}_{i-1}(t))-({\nu }_{i}+{\gamma }_{i}){Y}_{i}(t),\,i=1,2,3,\ldots ..,n-1,\\ 5.\,\frac{d{P}_{H}(t)}{dt} & = & H({Y}_{n-1}(t))-{\alpha }_{H}{P}_{H}(t),\\ 6.\,\frac{d{P}_{f}(t)}{dt} & = & \frac{{\beta }_{H}{\lambda }_{H}({P}_{H})[{S}_{H}(t)-1]}{{\Phi }_{H}[{I}_{H}(t)+1]}-({\alpha }_{f}+{\mu }_{f}){P}_{f}(t),\\ 7.\,\frac{d{X}_{j}(t)}{dt} & = & {f}_{j}({X}_{j-1}(t))-({\alpha }_{j}+{\mu }_{j}){X}_{j}(t),\,j=1,2,3,\ldots ..,m-1,\\ 8.\,\frac{d{P}_{h}(t)}{dt} & = & h({X}_{m-1}(t))-({\alpha }_{h}+{\mu }_{h}){P}_{h}(t).\end{array}$$

In the general multiscale model (1), the transmission rate of the environmentally transmitted disease system of type I is modelled by the expression *β*_*H*_*λ*_*H*_(*P*_*H*_). The function *λ*_*H*_ has the following specifications: *λ*_*H*_: [0, ∞] → [0, 1] represents the probability that a host is infected when exposed to environmental infectious reservoir *P*_*H*_. Further, the function *λ*_*H*_ must have the following two desirable properties^16^:

i. Property I: The probability of infection vanishes in absence of pathogen [i.e., *λ*_*H*_(0) = 0] and approach 1 as the environmental pathogen load becomes large [i.e., ${\mathrm{lim}}_{{P}_{H}\to \infty }\,{\lambda }_{H}({P}_{H})=1$];
ii. Property II: The probability of infection *λ*_*H*_(*P*_*H*_) increases with the environmental pathogen population *P*_*H*_, that is, ${\lambda ^{\prime} }_{H}({P}_{H}) > 0$, where prime denotes derivative with respect to the argument.
  Several possible functions *λ*_*H*_(*P*_*H*_) can satisfy the above listed two properties including the following two classic examples:
  - *The negative exponential infectivity response function*: This function has the form^16^
    2 $${\lambda }_{H}({P}_{H})=1-{e}^{-{\sigma }_{H}{P}_{H}}.$$
  - *The sigmoid infectivity response function*: This function has the form^17^

3 $${\lambda }_{H}({P}_{H})=\frac{{P}_{H}^{k}}{{P}_{0}^{k}+{P}_{H}^{k}},\,\,\,k=1,2,\ldots .,n,$$

where the parameter *k* defines the steepness of the sigmoid infectivity response.

In (a) and (b) above, *P*_0_ is a quantity of environmental pathogen load which gives 50% probability of infection and ${\sigma }_{H}=\,\mathrm{ln}\,(\frac{2}{{P}_{0}})$ ^18^. However, the multiscale modelling approach for environmentally transmitted infectious disease systems presented in this study is still in its foundation stages. Because of this, data collection on environmentally transmitted infectious disease systems are still not yet available to select a specific analytic form of ${\lambda }_{H}(.)$ with properties I and II on the basis of empirical evidence. Therefore, our selection of the analytic form of ${\lambda }_{H}(.\,)$ is limited to them being able to satisfy property I and property II above.

In the general multiscale model (1) for environmentally transmitted infectious disease systems of type I, Eqs (1) and (2) describe the transmission of an environmentally transmitted infectious disease system of type I at between-host scale where the force of infection is specified based on infectivity response functions (2) or (3) in terms of the pathogen load in the environment given by Eq. (5) of the general multiscale model (1). Equations (3–5) of the general multiscale model (1) describe the dynamics of the various pathogen populations in the different life stages in the outside-host environment. In these equations, Eq. (3) describes the dynamics of the pathogen population in the first life stage in the outside-host environment, while Eq. (5) describes changes in the pathogen population in the last life stage in the outside-host environment. Equation (4) describe the pathogen population dynamics in the intermediate life stages in the outside-host environment (i.e. at between-host scale). In all these equations (i.e. (3–5)), the transition from one life stage to another (modeled by *F*_*i*_(*Y*_*i*−1_(*t*)), *i* = 1, 2, …, *n* − 1, and *H*(*X*_*n*−1_(*t*)) is either through developmental changes to the next life stage or through production of the next life stage from the previous life stage that may involve the replication of the pathogen in the outside-host environment. The last three equations in the general multiscale model (1), that is, Eqs (6–8) describe the dynamics of the pathogen population in the different life stages in the inside-host environment i.e. at within-host scale. In these equations, Eq. (6) describes the dynamics of the pathogen population in the first life stage in the inside-host environment, while Eq. (8) describes changes in the pathogen population in the last life stage in the inside-host environment. Equation (7) describe pathogen population dynamics in the intermediate life stages in the inside-host environment. In the inside-host environment pathogen transition from one life stage to another (modeled by *f*_*j*_(*X*_*j*−1_(*t*)), *j* = 1, 2, …, *m* − 1, and *h*(*X*_*m*−1_(*t*)) is only through developmental changes, and no pathogen replication takes place in the inside-host environment as expected of environmentally transmitted infectious disease systems of type I. The variables of the general multiscale model (1) are summarized in Table 1.

**Table 1:** A summary of the variables of the general multiscale model (1).

No.	Variable	Description
1.	*S*_*H*_(*t*)	Population of susceptible humans at time t
2.	*I*_*H*_(*t*)	Population of infected humans at time t
3.	*P*_*F*_(*t*)	Population of the first life stage of the outside-host scale environmental pathogen at time t
4.	*P*_*H*_(*t*)	Population of the last life stage of the outside-host scale environmental pathogen at time t
5.	*Y*_*i*_(*t*), *i* = 1, 2, 3, …. *n* − 1	Populations of the intermediate life stages of the outside-host scale environmental pathogen which are not the first and last life stages at time t
6.	*P*_*f *_(*t*)	Population of the first life stage of the inside-host scale environmental pathogen at time t
7.	*P*_*h*_(*t*)	Population of the last life stage of the inside-host scale environmental pathogen at time t
8.	*X*_*j*_(*t*), *j* = 1, 2, 3, …. *m* − 1	Populations of the intermediate life stages of the inside-host scale environmental pathogen which are not the first and last life stages at time t

Following^7^, we can easily derive two important results which are:

- For positive parameters, the variables of the general multiscale model (1) with positive initial conditions will remain non-negative for all *t* ≥ 0 and for Λ_*H*_ > *μ*_*H*_, so that they do not violate a basic property of biological reality.
- For a specified *λ*_*H*_(*P*_*H*_) chosen from the possible list of infectivity response functions (2) and (3), the solutions of the general multiscale model (1) are bounded for Λ_*H*_ > *μ*_*H*_.

Therefore, the general multiscale model (1) is mathematically and biologically well-posed for Λ_*H*_ > *μ*_*H*_. We shall assume in all that follows (unless stated otherwise) that Λ_*H*_ > *μ*_*H*_.

## Application of the General Multiscale Model to Hookworm Infection As a Paradigm

**Figure 3:**
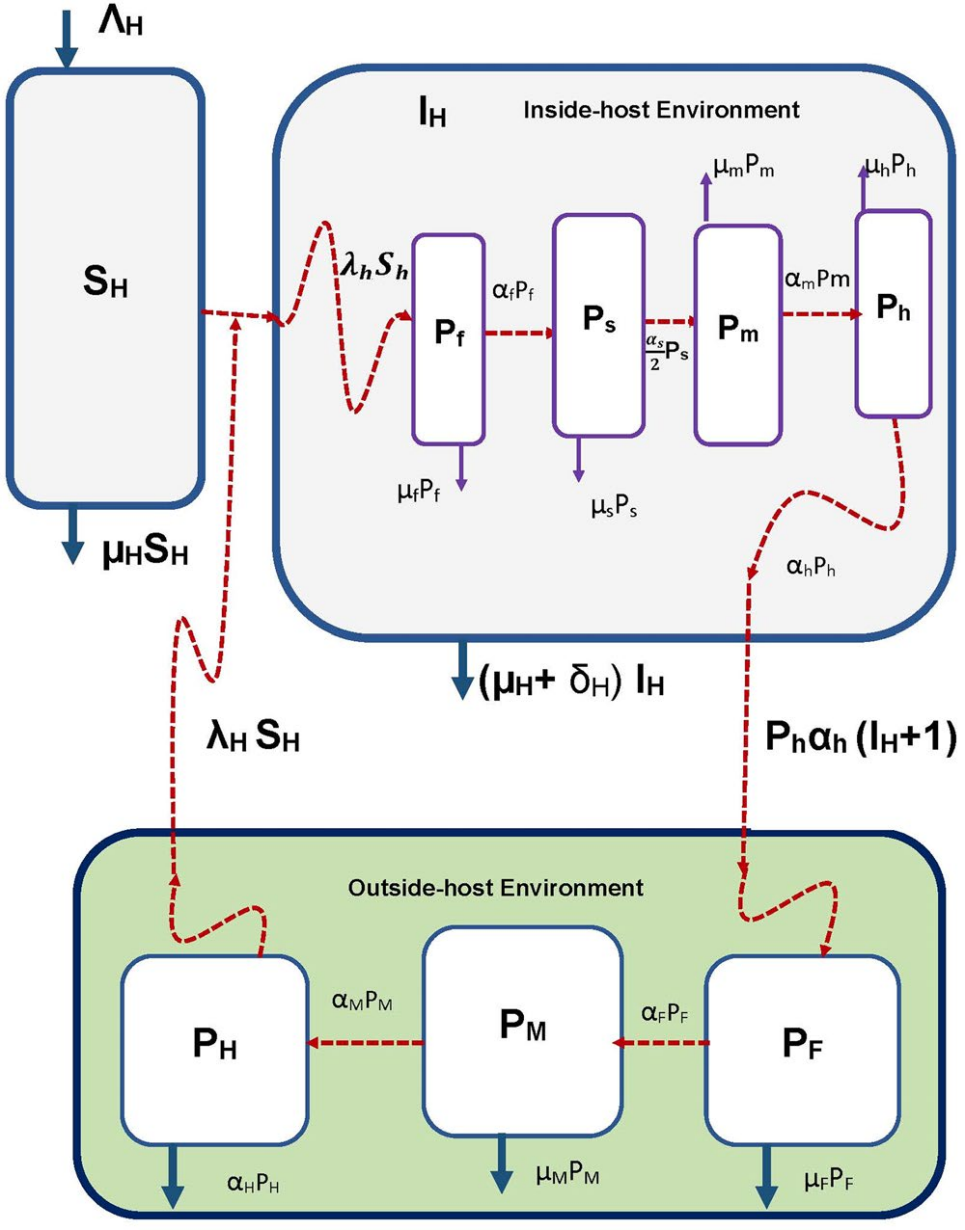
A conceptual diagram of the multiscale model of hookworm infectious disease system. In this Figure ${\lambda }_{h}{S}_{h}=\frac{{\beta }_{H}{P}_{H}(t)[{S}_{H}(t)-1]}{[{P}_{0}+{P}_{H}(t)]{\Phi }_{H}[{I}_{H}(t)+1]}$.

Hookworm infectious disease system is caused by a soil transmitted nematode called hookworm. This disease is of public health importance because as many as a billion people throughout the world’s tropical regions are infected by this intestinal parasite^19^. The two species that account for almost all human infections are (i) *Ancylostoma duodenale* – which is transmitted by both skin penetration as well as oral ingestion and predominates in more temperate zones, and (ii) *Necator americanus* – which is transmitted by oral ingestion only and is adapted to tropical conditions. The life cycle of hookworm has seven main stages – four of which occur in the human (i.e. at microscale) and the other three are in the geographical environment (i.e. at macroscale). For more information about the life cycle of hookworm the published paper^20^ provide more details. Only a brief description relevant for the multiscale modelling is provided in this section. In this section, we apply the general multiscale model of environmentally transmitted infectious disease of type I developed in the previous section to hookworm infection and explicitly incorporate the seven life stages of hookworm into the multiscale model. Figure 3 shows a conceptual diagram of the multiscale model of hookworm infection that explicitly incorporates the seven life stages of hookworm in terms of parasite population dynamics (i.e. pathogen load in each life stage). The first of the four life stages of hookworm in the human host (i.e. at microscale), denoted *X*_0_ = *P*_*f*_ in Fig. 3, in terms of parasite load in this life stage, begins with super-infection (i.e. repeated infection before the host recovers from an infectious episode). This involves movement of hookworm in its last life (denoted *Y*_*m*_ = *P*_*H*_ in Fig. 3 in terms of pathogen load in this life stage), in the geographical environment (i.e. at macroscale or the between-host scale), which is the infective life stage to the human host (known as the third stage larvae or *L*_3_) living in the soil. Following host entry, this first life stage migrates to the gastrointestinal tract. Within the gastrointestinal, this first life stage may either die naturally at an assumed rate of *μ*_*f*_, or molt at an assumed rate *α*_*f*_, to form the second life stage or first intermediate life stage which in Fig. 3 is denoted *X*_1_ = *P*_*s*_, in terms of the pathogen load in this life stage. In this second life stage *P*_*s*_ is reduced either by natural death at an assumed rate *μ*_*s*_, or by undergoing a second molt at a rate *α*_*s*_/2 to establish the population of the second intermediate life stage of hookworm denoted by *X*_2_ = *P*_*m*_ in Fig. 3, which is the population of mature worms assumed to have a natural death rate of *μ*_*m*_. The 1/2 multiplying *α*_*s*_ models the assumption that half of the immature worms are females and that we only consider developmental rate of female worms to sexual maturity in disease dynamics which give rise to final life stage of hookworm at within-host scale, which are the hookworm eggs. The female worms lay eggs which are the last life stage of hookworm at within-host scale. The population of worm eggs at within-host scale, denoted *X*_*m*_ = *P*_*h*_ in Fig. 3, either degrades at an assumed rate *μ*_*h*_ or is excreted/shed into the geographical environment (i.e. the macroscale) at an assumed rate *α*_*h*_. The shedding/excretion of hookworm eggs establishes the first life stage of hookworm at between-host scale. This first life stage of hookworm at between-host scale (denoted *Y*_0_ = *P*_*F*_ in Fig. 3), may deplete through degrading of the eggs in the macroscale environment at an assumed rate *μ*_*F*_, or the eggs may hatch at an assumed rate *α*_*F*_ to give rise to the first and last intermediate life stage called the first-stage larvae or *L*_1_, (also called rhabitiform), denoted denoted *Y*_1_ = *P*_*M*_ in Fig. 3. The *L*_1_ molts at an assumed rate of *α*_*M*_ to give rise to the last life stage of hookworm at between-host scale called the third-stage larvae or *L*_3_, which is infectious to the human host which initiates the hookworm reproductive cycle again by establishing its first life stage in the human host through super-infection. This last and infectious life stage of hookworm, denoted *Y*_*n*_ = *P*_*H*_ in Fig. 3 in terms of the pathogen load in this life stage may be depleted in the geographical environment by having an average life span of 1/*α*_*H*_. If we choose ${\lambda }_{H}(t)=\frac{{\beta }_{H}{P}_{H}(t)}{{P}_{0}+{P}_{H}(t)}$ from the infectivity response functions (2) and (3) specified for the general multiscale model (1) in section 4, then the super-infection that introduces the population of first life stage of hookworm at the within-host scale (denoted *X*_0_ = *P*_*f*_, is modelled by $\frac{{\beta }_{H}{\lambda }_{H}({P}_{H})[{S}_{H}(t)-1]}{{\Phi }_{H}[{I}_{H}(t)+1]}=\frac{{\beta }_{H}{P}_{H}(t)[{S}_{H}(t)-1]}{[{P}_{0}+{P}_{H}(t)]{\Phi }_{H}[{I}_{H}(t)+1]}$. Taking into account all these specifications and descriptions, the multiscale model for hookworm infection (which is also represented in conceptual form in Fig. 3) becomes:

4 $$\begin{array}{ccc}1.\frac{d{S}_{H}(t)}{dt} & = & {\Lambda }_{H}-\frac{{\beta }_{H}{P}_{H}(t){S}_{H}(t)}{{P}_{0}+{P}_{H}(t)}-{\mu }_{H}{S}_{H}(t),\\ 2.\frac{d{I}_{H}(t)}{dt} & = & \frac{{\beta }_{H}{P}_{H}(t){S}_{H}(t)}{{P}_{0}+{P}_{H}(t)}-({\mu }_{H}+{\delta }_{H}){I}_{H}(t),\\ 3.\frac{d{P}_{F}(t)}{dt} & = & [{I}_{H}(t)+1]{\alpha }_{h}{P}_{h}(t)-({\mu }_{F}+{\alpha }_{F}){P}_{F}(t),\\ 4.\frac{d{P}_{M}(t)}{dt} & = & {\alpha }_{F}{P}_{F}(t)-({\mu }_{M}+{\alpha }_{M}){P}_{M}(t),\\ 5.\frac{d{P}_{H}(t)}{dt} & = & {\alpha }_{M}{P}_{M}(t)-{\alpha }_{H}{P}_{H}(t),\\ 6.\frac{d{P}_{f}(t)}{dt} & = & \frac{{\beta }_{H}{P}_{H}(t)[{S}_{H}(t)-1]}{[{P}_{0}+{P}_{H}(t)]{\Phi }_{H}[{I}_{H}(t)+1]}-({\mu }_{f}+{\alpha }_{f}){P}_{f}(t),\\ 7.\frac{d{P}_{s}(t)}{dt} & = & {\alpha }_{f}{P}_{f}(t)-({\mu }_{s}+{\alpha }_{s}){P}_{s}(t),\\ 8.\frac{d{P}_{m}(t)}{dt} & = & \frac{{\alpha }_{s}}{2}{P}_{s}(t)-{\mu }_{m}{P}_{m}(t),\\ 9.\frac{d{P}_{h}(t)}{dt} & = & {\alpha }_{m}{P}_{m}(t)-({\mu }_{h}+{\alpha }_{h}){P}_{h}(t).\end{array}$$

The parameters of the multiscale model (4) are summarized in Table 2. However, not all modelling is without limitations. One of the limitations of the multiscale model (4) is that parameters used to evaluate it could not be chosen from a single epidemic situation. The demographic parameters marked [H^*^] were chosen to be within ranges of values for developing countries. Similarly, where epidemiological parameters specific to hookworm infection could not be found, parameters marked [R^*^] were used. The parameters marked [R^*^] were chosen to be within ranges of values for helminths infections. A further limitation of the multiscale modelling approach in this article is that it does not incorporate heterogeneity and other realistic features of the ecology and evolution of infectious disease systems such as selection, mutation and the development of immune response to a pathogen which is a form of co-evolution in the host-pathogen interaction. Therefore, the multiscale modelling approach in this article offers opportunities for extensions by taking advantage of the multiscale vision in this article to develop individual-based multiscale models (IMSMs)^3,4^ such as (i) network-based multiscale models - which are developed using graph theoretic or network modelling techniques, or (ii) data-based multiscale models (D-MSMs) - which are developed using statistical modelling techniques to model hierarchical empirical data, or (iii) simulation-based multiscale models (S-MSMs) - which are based on computational algorithms which include agent-based models (ABM), cellular automata (CA) and petri-nets (PN) to account for heterogeneity, mutation, selection, etc. in multiscale modelling of infectious disease systems.

**Table 2:** Table of parameter values for the multiscale model given by (4).

No.	Para-meter	Meaning	Value [Range explored]	Units	Source/Rational
1.	Λ_*H*_	Susceptible humans recruitment rate through birth and immigration	0.0001 [0.0001–0.0003]	*day* ^−1^	[H^*^]
2.	*β*_*H*_	Human infection rate	0.1 [0.3000–0.00300]	*day* ^−1^	[R^*^]
3.	*μ*_*H*_	Natural death rate of humans	0.00001 [0.0001–0.00001]	*day* ^−1^	[H^*^]
4.	*δ*_*H*_	Disease induced death rate	0.004 [0.004–0.0001]	*day* ^−1^	[H^*^]
5.	*P*_0_	Saturation constant of hookworm infection	1000 [100–10000]	*day* ^−1^	assumed
6.	*α*_*H*_	Natural decay of infective larvae	0.2000 [0.1000–0.3333]	*day* ^−1^	^25^
7.	*α*_*F*_	Rate at which eggs hatch	0.7000 [0.5000–1.000]	*day* ^−1^	^26^
8.	*μ*_*F*_	Natural decay rate of hookworm eggs in the geographical environment	0.183561 [0.079472–0.260274]	*day* ^−1^	^9^
9.	Φ_*H*_	Proportion of new infections	0.03000 [0.003–0.3000]	*day* ^−1^	assumed
10.	*α*_*M*_	Rate at which immature worms become infective worms	0.200 [0.0714–0.500]	*day* ^−1^	^27^
11.	*μ*_*M*_	Natural death of immature worms in the geographical environment	1.427397 [0.805479–2.276712]	*day* ^−1^	^9^
12.	*μ*_*f*_	Natural death rate of first life stage hookworm in human host	0.0400 [0.0200–0.0600]	*day* ^−1^	[R^*^]
13.	*α*_*f*_	Migration rate of first life stage hookworm to small intestine	0.0250 [0.0200–0.0357]	*day* ^−1^	[R^*^]
14.	*μ*_*s*_	Natural death rate of immature worms in the small intestine	0.0400 [0.02–0.0800]	*day* ^−1^	[R^*^]
15.	*α*_*s*_	Developmental rate to matured worms	0.0250[0.0200–0.0357]	*day* ^−1^	^28^
16.	*α*_*m*_	Rate at which adult female worm produces eggs	10000.0 [3000.0–20000.0]	*day* ^−1^	^25^
17.	*μ*_*m*_	Natural death rate of mature female worms within the human host	0.000833 [0.000685–0.000913]	*day* ^−1^	^28^
18.	*α*_*h*_	Rate of excretion of the hookworm eggs into the geographical environment	0.320548 [0.019178–1.369863]	*day* ^−1^	^29, 30^
19.	*μ*_*h*_	Natural decay of hookworm eggs in the human host	0.0400 [0.0400–0.0001]	*day* ^−1^	[R^*^]

## Evidence for the Reciprocal Influence Between the Macroscale and the Microscale from Analysis of the Multiscale Model

In this section we present two pieces of evidence about the reciprocal influence between the microscale and the macroscale which we obtain from the analysis of the multiscale model (4). The two pieces of evidence are derived from analysis of the reproductive number and the endemic equilibrium of the multiscale model (4) in the following two sub-sections.

### Evidence of the reciprocal influence between the macroscale and the microscale from reproductive number

We determine the basic reproductive number for the multiscale model (4) by using the next generation operator approach^21^. Following the approach in^21^, the multiscale model (4) can be written in the form

5 $$\begin{array}{ccc}\frac{dX}{dt} & = & f(X,Y,Z),\\ \frac{dY}{dt} & = & g(X,Y,Z),\\ \frac{dZ}{dt} & = & h(X,Y,Z),\end{array}$$

where,

i. *X* = (*S*_*H*_) represents all compartments of individuals that are not infected,
ii. *Y* = (*I*_*H*_, *P*_*f*_, *P*_*s*_, *P*_*m*_, *P*_*h*_, *P*_*F*_, *P*_*M*_) represents all compartments of infected individuals that are not capable of infecting others,
iii. *Z* = (*P*_*H*_) represents all compartments of infected individuals who are capable of infecting others.

Following the other additional steps in^21^, the basic reproductive number of the multiscale model (4) can be shown to be,

6 $$\begin{array}{ccc}{{\mathscr{R}}}_{0} & = & {{\mathscr{R}}}_{0h}{{\mathscr{R}}}_{0H},\\  & = & \frac{1}{2}.\frac{{\alpha }_{m}}{{\mu }_{m}}.\frac{{\alpha }_{h}}{({\mu }_{h}+{\alpha }_{h})}.\frac{{\alpha }_{s}}{({\mu }_{s}+{\alpha }_{s})}.\frac{{\alpha }_{f}}{({\mu }_{f}+{\alpha }_{f})}.\frac{{\alpha }_{F}}{({\mu }_{F}+{\alpha }_{F})}\\  &  & \times \,.\frac{{\alpha }_{M}}{({\mu }_{M}+{\alpha }_{M})}.\frac{{\beta }_{H}({\Lambda }_{H}-{\mu }_{H})}{{\mu }_{H}({\mu }_{H}+{\delta }_{H}){\Phi }_{H}{\alpha }_{H}{P}_{0}}.\end{array}$$

In the expression for ${ {\mathcal R} }_{0}$ given (6), ${ {\mathcal R} }_{0h}$ is the within-host scale partial reproductive number while and *R*_0*H*_ is the between-host scale partial reproductive number given by

7 $$\begin{array}{ccc}{{\mathscr{R}}}_{0h} & = & \frac{1}{2}.\frac{{\alpha }_{m}}{{\mu }_{m}}.\frac{{\alpha }_{h}}{({\mu }_{h}+{\alpha }_{h})}.\frac{{\alpha }_{s}}{({\mu }_{s}+{\alpha }_{s})}.\frac{{\alpha }_{f}}{({\mu }_{f}+{\alpha }_{f})},\\ {{\mathscr{R}}}_{0H} & = & \frac{{\alpha }_{F}}{({\mu }_{F}+{\alpha }_{F})}.\frac{{\alpha }_{M}}{({\mu }_{M}+{\alpha }_{M})}.\frac{{\beta }_{H}({\Lambda }_{H}-{\mu }_{H})}{{\mu }_{H}({\mu }_{H}+{\delta }_{H}){\Phi }_{H}{\alpha }_{H}{P}_{0}}.\end{array}$$

We conclude from the expression (6) of the basic reproductive number of hookworm infection that it is a function of both the within-host scale parameters and the between-host scale parameters. Therefore, the obtained results here show that the microscale (within-host scale) and the macroscale (between-host scale) influence each other in a reciprocal way for hookworm infection.

### Evidence of the reciprocal influence between the macroscale and the microscale from endemic equilibrium

Here we look at the endemic equilibrium of the multiscale model (4) to establish evidence for the reciprocal influence between the within-host scale and the between-host scale. Let

8 $${E}^{\ast }=({S}_{H}^{\ast },{I}_{H}^{\ast },{P}_{f}^{\ast },{P}_{s}^{\ast },{P}_{m}^{\ast },{P}_{h}^{\ast },{P}_{F}^{\ast },{P}_{M}^{\ast },{P}_{H}^{\ast })$$

be an endemic solution for the multiscale model (4). We then express ${S}_{H}^{\ast },{I}_{H}^{\ast },{P}_{f}^{\ast },{P}_{s}^{\ast },{P}_{m}^{\ast },{P}_{h}^{\ast },{P}_{F}^{\ast },{P}_{M}^{\ast }$ in terms of ${P}_{H}^{\ast }$ as follows:

9 $$\begin{array}{ccc}{S}_{H}^{\ast }({P}_{H}^{\ast }) & = & \frac{{\Lambda }_{H}({P}_{0}+{P}_{H}^{\ast })}{{\mu }_{H}{P}_{0}+({\beta }_{H}+{\mu }_{H}){P}_{H}^{\ast }},\,{I}_{H}^{\ast }({P}_{H}^{\ast })=\frac{1}{({\mu }_{H}+{\delta }_{H})}.\frac{{\beta }_{H}{\Lambda }_{H}{P}_{H}^{\ast }}{[{\beta }_{H}{P}_{H}+{\mu }_{H}({P}_{0}+{P}_{H})]},\\ {P}_{f}^{\ast }({P}_{H}^{\ast }) & = & \frac{1}{({\mu }_{f}+{\alpha }_{f})}.\frac{{Q}_{M}^{\ast }({P}_{H}^{\ast })}{({I}_{H}^{\ast }+1)},\,{Q}_{M}^{\ast }({P}_{H}^{\ast })=\frac{{\beta }_{H}{P}_{H}^{\ast }[({\Lambda }_{H}-{\mu }_{H})({P}_{0}+{P}_{H}^{\ast })-{\beta }_{H}{P}_{H}^{\ast }]}{({P}_{0}+{P}_{H}^{\ast }){\Phi }_{H}[{\beta }_{H}{P}_{H}^{\ast }+{\mu }_{H}({P}_{0}+{P}_{H}^{\ast })]},\\ {P}_{m}^{\ast }({P}^{\ast }) & = & \frac{1}{2}.\frac{1}{{\mu }_{m}}.\frac{{\alpha }_{s}}{({\mu }_{s}+{\alpha }_{s})}.\frac{\alpha }{({\mu }_{f}+{\alpha }_{f})}.\frac{{Q}_{M}^{\ast }({P}_{H}^{\ast })}{({I}_{H}^{\ast }+1)},\\ {P}_{s}^{\ast }({P}^{\ast }) & = & \frac{1}{({\mu }_{f}+{\alpha }_{f})}.\frac{{\alpha }_{f}}{({\mu }_{f}+{\alpha }_{f})}.\frac{{Q}_{M}^{\ast }({P}_{H}^{\ast })}{({I}_{H}^{\ast }+1)},\\ {P}_{h}^{\ast }({P}^{\ast }) & = & \frac{1}{2}.\frac{1}{({\mu }_{h}+{\alpha }_{h})}.\frac{{N}_{m}{\alpha }_{m}}{{\mu }_{m}}.\frac{{\alpha }_{s}}{({\mu }_{s}+{\alpha }_{s})}.\frac{\alpha }{({\mu }_{f}+{\alpha }_{f})}.\frac{{Q}_{M}^{\ast }({P}_{H}^{\ast })}{({I}_{H}^{\ast }+1)},\\ {P}_{F}^{\ast }({P}_{P}^{\ast }) & = & \frac{1}{2}.\frac{1}{({\mu }_{F}+{\alpha }_{F})}.\frac{{\alpha }_{h}}{({\mu }_{h}+{\alpha }_{h})}.\frac{{N}_{m}{\alpha }_{m}}{{\mu }_{m}}.\frac{{\alpha }_{s}}{({\mu }_{s}+{\alpha }_{s})}.\frac{\alpha }{({\mu }_{f}+{\alpha }_{f})}.{Q}_{M}^{\ast }({P}_{H}^{\ast }),\\ {P}_{M}^{\ast }({P}_{H}^{\ast }) & = & \frac{1}{2}.\frac{{\alpha }_{M}}{({\mu }_{M}+{\alpha }_{M})}.\frac{{\alpha }_{F}}{({\mu }_{F}+{\alpha }_{F})}.\frac{{\alpha }_{h}}{({\mu }_{h}+{\alpha }_{h})}.\frac{{N}_{m}{\alpha }_{m}}{{\mu }_{m}}.\frac{{\alpha }_{s}}{({\mu }_{s}+{\alpha }_{s})}.\frac{\alpha }{({\mu }_{f}+{\alpha }_{f})}.{Q}_{M}^{\ast }({P}_{H}^{\ast }).\end{array}$$

Substitute Eq. (9) in the equation for *P*_*H*_ which is given by:

10 $$\frac{d{P}_{H}(t)}{dt}={\alpha }_{M}{P}_{M}(t)-{\alpha }_{H}{P}_{H}(t),$$

at endemic equilibrium we get

11 $${P}_{H}^{\ast 2}+B{P}_{H}^{\ast }+C=0,$$

where

12 $$B=\frac{{\alpha }_{M}}{{\alpha }_{M}+{\mu }_{M}}.\frac{{\alpha }_{F}}{{\alpha }_{F}+{\mu }_{F}}.\frac{{ {\mathcal R} }_{0h}{\beta }_{H}^{2}}{{\Phi }_{H}{\alpha }_{H}({\mu }_{H}+{\beta }_{H})}+{P}_{0}-\frac{{\mu }_{H}{P}_{0}({ {\mathcal R} }_{0}-1)}{({\mu }_{H}+{\beta }_{H})},$$

13 $$C=-\,\frac{{\mu }_{H}{P}_{0}^{2}({ {\mathcal R} }_{0}-1)}{({\mu }_{H}+{\beta }_{H})}.$$

Therefore,

14 $${P}_{H}^{\ast }=\frac{1}{2}[-\,B+\sqrt{{B}^{2}-4C}] > 0\,{\rm{for}}\,{ {\mathcal R} }_{0} > 1.$$

Note that $C < 0$ for ${ {\mathcal R} }_{0} > 1$ while B is either positive or negative for ${ {\mathcal R} }_{0} > 1$. Then we conclude that the multiscale model (4) has one positive endemic equilibrium for ${ {\mathcal R} }_{0} > 1$. Further, we deduce that the between-host scale endemic expressions for ${S}_{H}^{\ast },{I}_{H}^{\ast },{P}_{F}^{\ast },{P}_{M}^{\ast },{P}_{H}^{\ast }$ are determined by both within-host scale disease parameters and between-host scale parameters and in turn, the within-host scale endemic expressions for ${P}_{f}^{\ast },{P}_{s}^{\ast },{P}_{m}^{\ast },{P}_{h}^{\ast }$ are dependent on both within-host scale and between-host scale parameters. Therefore, the obtained results here also show that the microscale (within-host scale) and the macroscale (between-host scale) influence each other in a reciprocal way.

## Evidence of Reciprocal Influence Between Microscale and Macroscale from Numerical Simulations of the Multiscale Model

In this section, we present evidence for the reciprocal influence between the macroscale and the microscale using results from the numerical simulations of the multiscale model (4). The simulations for the multiscale model (4) are presented in the two sub-sections that follow. The initial conditions used for the simulations of multiscale model (4) are: ${S}_{H}(0)=20000$, ${I}_{H}(0)=0$, ${P}_{F}(0)=0$, ${P}_{m}(0)=0$, ${P}_{H}(0)=200$, ${P}_{f}(0)=20$, ${P}_{m}(0)=0$, ${P}_{s}(0)=0$, and ${P}_{h}(0)=0$. The disease parameter values are presented in Table 2.

### Evidence of the influence of within-host scale parameters on between-host scale variables for the hookworm infection

**Figure 4:**
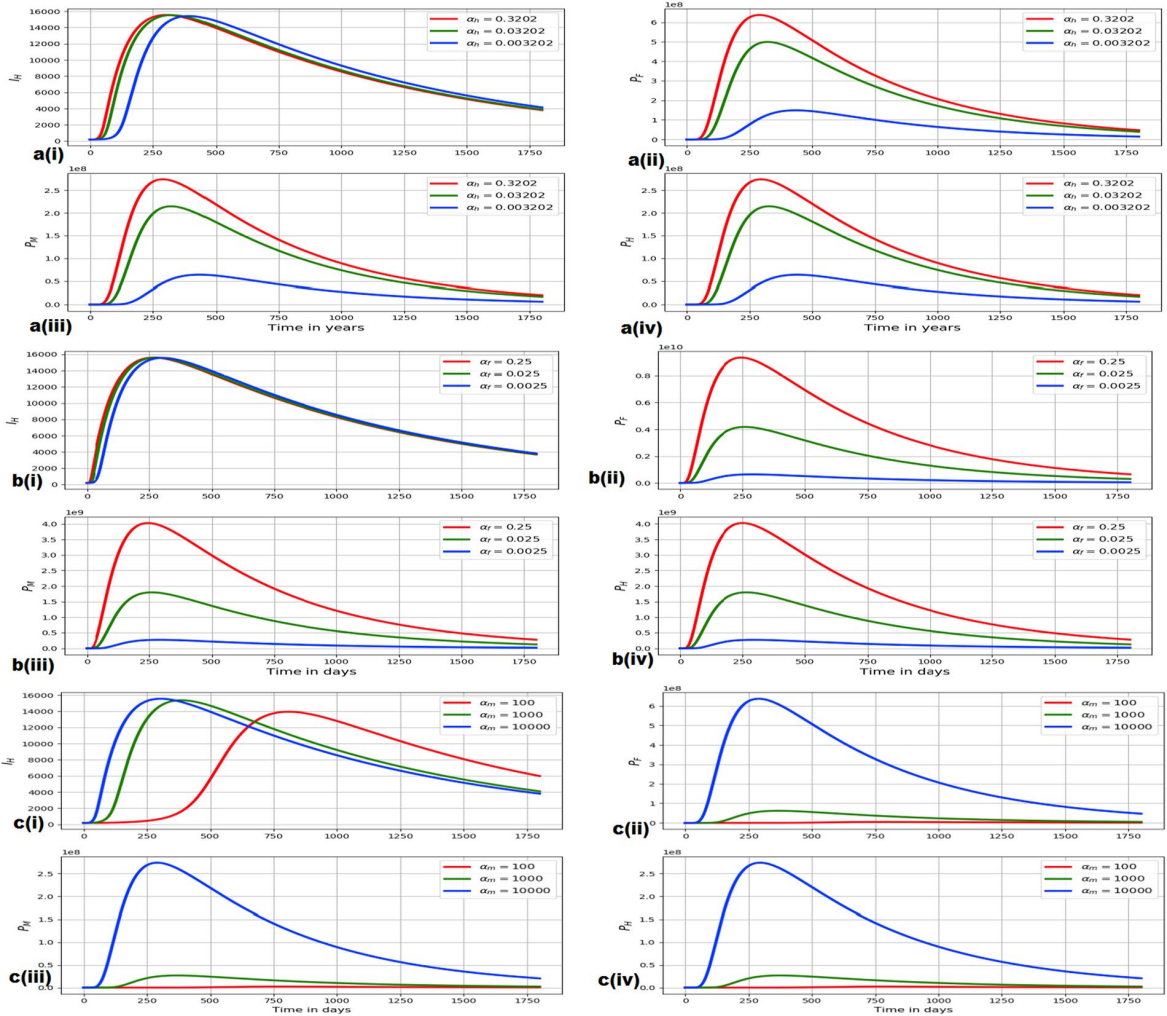
(**a**) Shows evolution of *I*_*H*_, *P*_*F*_, *P*_*M*_ and *P*_*H*_ for different values *α*_*h*_: *α*_*h*_ = 0.3202, *α*_*h*_ = 0.03202 and *α*_*h*_ = 0.003202. (**b**) Shows evolution of *I*_*H*_, *P*_*F*_, *P*_*M*_ and *P*_*H*_ for different values *α*_*f*_: *α*_*f*_ = 0.25, *α*_*f*_ = 0.025 and *α*_*f*_ = 0.0025. (**c**) Shows evolution of *I*_*H*_, *P*_*F*_, *P*_*M*_ and *P*_*H*_ for different values *α*_*m*_: *α*_*m*_ = 100, *α*_*m*_ = 1000 and *α*_*m*_ = 10000.

In this sub-section we present evidence for the influence of selected with-host scale parameters (*α*_*h*_, *α*_*f*_ and *α*_*m*_) on between-host scale variables (*I*_*H*_, *P*_*F*_, *P*_*M*_ and *P*_*H*_) using the multiscale model (4) for hookworm infection. The results of the influence of within-host scale parameters on between-host scale variables for the hookworm infection are shown in Fig. 4 as follows.

- *The influence of α*_*h*_ *on I*_*H*_, *P*_*F*_, *P*_*M*_ *and P*_*H*_: Fig. 4(a) shows evolution of *I*_*H*_, *P*_*F*_, *P*_*M*_ and *P*_*H*_ for different values *α*_*h*_: *α*_*h*_ = 0.3202, *α*_*h*_ = 0.03202 and *α*_*h*_ = 0.003202.
- *The influence of α*_*f*_ *on I*_*H*_, *P*_*F*_, *P*_*M*_ *and P*_*H*_: Fig. 4(b) shows evolution of *I*_*H*_, *P*_*F*_, *P*_*M*_ and *P*_*H*_ for different values *α*_*f*_: *α*_*f*_ = 0.25, *α*_*f*_ = 0.025 and *α*_*f*_ = 0.0025.
- *The influence of α*_*m*_ *on I*_*H*_, *P*_*F*_, *P*_*M*_ *and P*_*H*_: Fig. 4(c) shows evolution of *I*_*H*_, *P*_*F*_, *P*_*M*_ and *P*_*H*_ for different values *α*_*m*_: *α*_*m*_ = 100, *α*_*m*_ = 1000 and *α*_*m*_ = 10000.

From all the three sets of numerical results in Fig. 4 (i.e. Fig. 4(a–c)), we notice that as the within-host scale parameters *α*_*h*_, *α*_*f*_ and *α*_*m*_ change, the between-host scale variables *I*_*H*_, *P*_*F*_, *P*_*M*_ and *P*_*H*_ also change. Further, although the results shown in Fig. 4 are for a selected few within-host scale parameters to keep the article within reasonable size, other within-host scale parameters we investigated (whose results are not reported here) showed similar influence on between-host scale variables *I*_*H*_, *P*_*F*_, *P*_*M*_ and *P*_*H*_. This implies that during disease dynamics within-host scale influences between-host scale for hookworm infection.

### Evidence of the influence of between-host scale parameters on within-host scale variables for the hookworm infection

**Figure 5:**
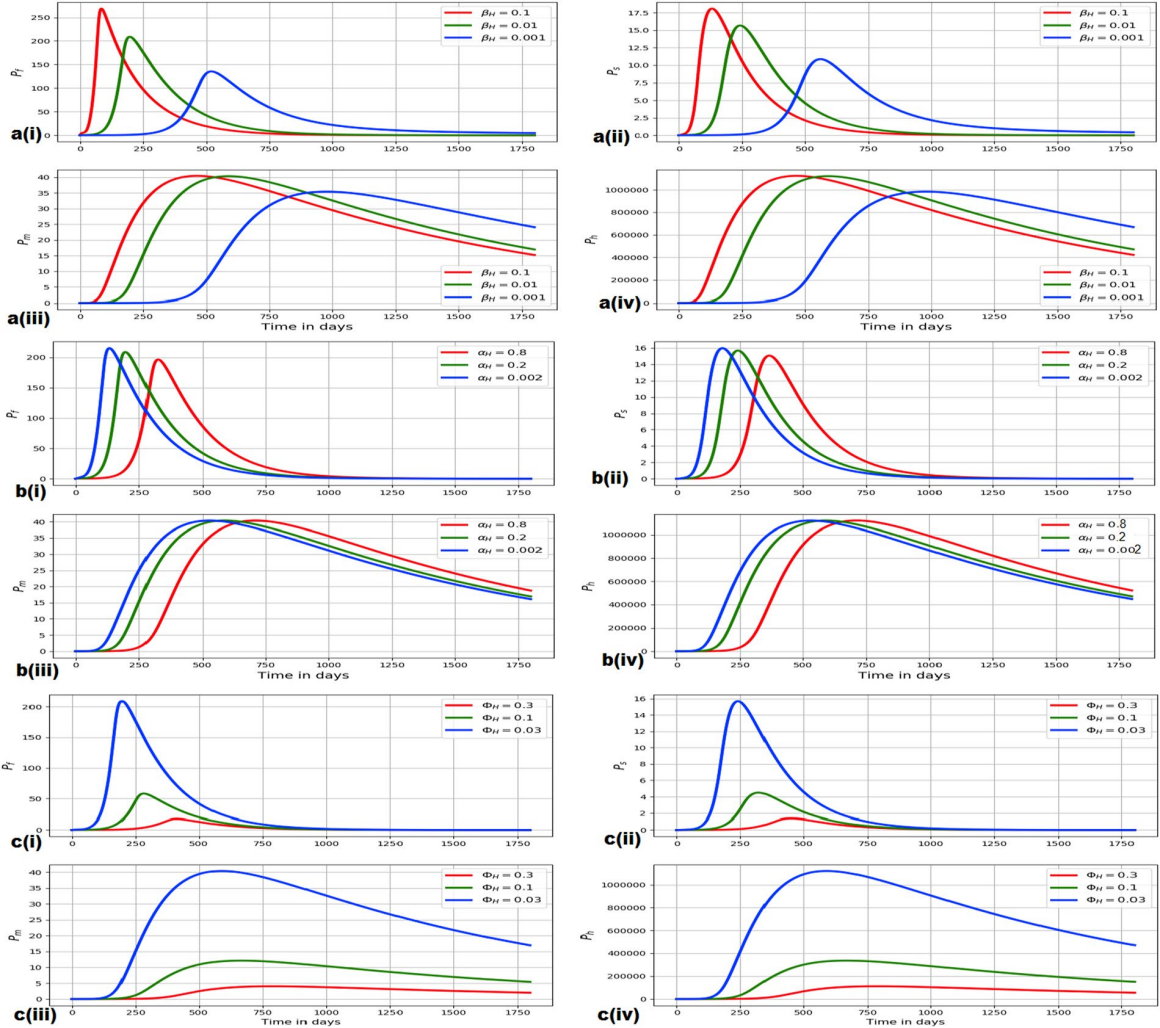
(**a**) Shows evolution of *P*_*f*_, *P*_*s*_, *P*_*m*_ and *P*_*h*_ for different values *β*_*H*_: *β*_*H*_ = 0.1, *β*_*H*_ = 0.01 and *β*_*H*_ = 0.001. (**b**) Shows evolution of *P*_*f*_, *P*_*s*_, *P*_*m*_ and *P*_*h*_ for different values *α*_*H*_: *α*_*H*_ = 0.8, *α*_*H*_ = 0.2 and *α*_*H*_ = 0.002. (**c**) Shows evolution of *P*_*f*_, *P*_*s*_, *P*_*m*_ and *P*_*h*_ for different values Φ_*H*_: Φ_*H*_ = 0.3, Φ_*H*_ = 0.1 and Φ_*H*_ = 0.03.

In this sub-section we present evidence for the influence of selected between-host scale parameters (*β*_*H*_, *α*_*H*_ and Φ_*H*_) on within-host scale variables (*P*_*f*_, *P*_*s*_, *P*_*m*_ and *P*_*h*_) using the multiscale model (4) for hookworm infection. The results of the influence of between-host scale parameters on within-host scale variables for the hookworm infection are shown in Fig. 5 as follows.

- *The influence of β*_*H*_ *on P*_*f*_, *P*_*s*_, *P*_*m*_ *and P*_*h*_: Fig. 5(a) shows evolution of *P*_*f*_, *P*_*s*_, *P*_*m*_ and *P*_*h*_ for different values *β*_*H*_: *β*_*H*_ = 0.1, *β*_*H*_ = 0.01 and *β*_*H*_ = 0.001.
- *The influence of α*_*H*_ *on P*_*f*_, *P*_*s*_, *P*_*m*_ *and P*_*h*_: Fig. 5(b) shows evolution of *P*_*f*_, *P*_*s*_, *P*_*m*_ and *P*_*h*_ for different values *α*_*H*_: *α*_*H*_ = 0.8, *α*_*H*_ = 0.2 and *α*_*H*_ = 0.002.
- *The influence of* Φ_*H*_ *on P*_*f*_, *P*_*s*_, *P*_*m*_ *and P*_*h*_: Fig. 5(c) shows evolution of *P*_*f*_, *P*_*s*_, *P*_*m*_ and *P*_*h*_ for different values Φ_*H*_: Φ_*H*_ = 0.3, Φ_*H*_ = 0.1 and Φ_*H*_ = 0.03.

From all the three sets of numerical results in Fig. 5 (i.e. Fig. 5(a–c)), we notice that as the between-host scale parameters *β*_*H*_, *α*_*H*_ and Φ_*H*_ change, the within-host scale variables *P*_*f*_, *P*_*s*_, *P*_*m*_ and *P*_*h*_ also change. Further, although the results shown in Fig. 5 are also for a selected few between-host scale parameters to keep the manuscript within reasonable size, other between-host scale parameters we investigated (whose results are not reported here) also showed similar influence on within-host scale variables *P*_*f*_, *P*_*s*_, *P*_*m*_ and *P*_*h*_. This implies that during disease dynamics between-host scale influences within-host scale for hookworm infection.

Collectively, the numerical results shown in Figs 4 and 5 show that during hookworm disease dynamics, there is reciprocal influence between the within-host scale (microscale) and the between-host scale (macroscale). These numerical results are in agreement with those obtained in section 6, where we showed that both the reproductive number and the endemic equilibrium for hookworm infection are functions of both the within-host scale parameters and between-host scale parameters. Therefore, the results in section 6 and this section are powerful evidence for the existence of reciprocal influence of the between-host scale and the within-host scale for hookworm infection.

## Discussion and Conclusions

This article explored the replication-transmission relativity theory which states that at any level of organization of an infectious disease system there is no privileged/absolute scale which would determine disease dynamics, only interactions between the microscale and macroscale. This replication-transmission relativity theory places multiscale modelling of infectious disease systems on sound theoretical foundations. The central idea of this replication-transmission relativity theory is that at any level of organization of an infectious disease system, there exists a pathogen replication-transmission multiscale loop which is sustained by (i) pathogen shedding/excretion - which involves movement of pathogen from the microscale to the macroscale, and thereby constituting the linkage of the microscale to the macroscale, and (ii) infection/super-infection by pathogen - which involves movement of pathogen from the macroscale to the microscale, and thereby constituting the linkage of the macroscale to the microscale. Therefore, the replication-transmission relativity theory presented in this article is a critically important framework for development of a wide variety of multiscale models at different hierarchical levels of organization of an infectious disease system. Evidence of the validity of the replication-transmission relativity theory in this article was presented using a multiscale model of hookworm infectious disease system developed at host level. Although the validity of this theory was illustrated using a multiscale model with the following traits: (i) it is a multiscale for a specific disease system, that is, hookworm infection, (ii) it is a a multiscale model for disease with a specific transmission mechanism, that is, an environmentally transmitted disease system, and (iii) it is a multiscale model developed at a specific level of organization of the infectious disease system, that is the host level, we think that the results obtained for hookworm infection can be generalized to imply that for infectious disease systems this reciprocal influence between the microscale and macroscale is some kind of replication-transmission relativity that can be considered as an extension of the relativity principle in physics by proposing that at any level of organization of an infectious disease system there is no privileged/absolute scale which would determine events at the other scale, only interactions between the microscale and macroscale because of the following considerations:

- Two recent papers^22,23^ established that environmentally transmitted and directly transmitted infectious disease systems can be modeled using the same approach at host level by using the concept of community pathogen as a new public health measure. In these papers the authors established a multiscale modelling science base for directly transmitted infectious disease systems (where the inside-host environment’s biological entities such as cells, tissues, organs, body fluids, whole body are the reservoir of infective pathogen in the community) that is comparable to the multiscale modelling science base for environmentally transmitted infectious disease systems presented in this article (where the outside-host geographical environment’s physical entities such as soil, air, formites/contact surfaces, food and water are the reservoir of infective pathogen in the community). They achieved this by defining a new public health measure called community pathogen load (see^22,23^ for details), which is then used to define the force of infection. This community pathogen load is comparable in meaning to environmental pathogen load and the approach can be used to convert transmission models for directly transmitted infectious disease systems which are developed by compartmentalizing the population (cells, tissues, hosts, etc.) into susceptible, exposed, infected, recovered (SEIR), and variations of this paradigm (SI, SIS, SEI, SEIS, SIR, SIRS, SEIRS, etc.) into equivalent transmission models for environmentally transmitted infectious disease systems which are developed by compartmentalizing the population (cells, tissues, hosts, etc.) into susceptible, exposed, infected, recovered, and environmental pathogen load (SEIRP), and variations of this paradigm (SIP, SISP, SEIP, SEISP, SIRP, SIRSP, SEIRSP, etc.). The community pathogen load like the environmental pathogen load is also a measure of community-wide pool of infectious reservoir, and is derived by making the assumption that hosts are small homogeneous habitats/environments in the community in which pathogens multiply and grow to become infectious. These inside-host environments (which are the only habitat for directly transmitted pathogens) for which community pathogen load is a measure of community-wide disease transmission, are considered to play a similar role to outside-host environments (which are also habitat for environmentally transmitted pathogens). This implies that the multiscale modelling vision in this article can be extended to directly transmitted infectious disease systems to illustrate the reciprocal influence between the microscale and the macroscale at host level.
- Infectious disease systems are different from other complex systems in that the same thing happens at every level of organization of an infectious disease system. In most complex systems each level of organization usually requires a separate analysis because it is usually the case that mechanisms and processes of the system at each level are different. However, the mechanisms and process (transmission, replication, pathogen shedding, infection/super-infection) for infectious disease systems are the same for each hierarchical level of multiscale observation of an infectious disease system, leading to similar categories of multiscale models that describe infectious disease dynamics at all the main levels of organization of an infectious disease system which include^3,4^: (i) individual-based multiscale models (IMSMs), (ii) nested multiscale models (NMSMs), (iii) embedded multiscale models (EMSMs), (iv) hybrid multiscale models (HMSMs), and (v) coupled multiscale models. These multiscale models describe invariant relationships between the microscale and the macroscale, as prescribed by the replication-transmission relativity within successive levels of organization of an infectious disease system.

Collectively these two arguments imply that the replication-transmission relativity theory’s validity is not restricted to a specific infectious disease system, or a specific infectious disease systems with a specific transmission mechanism or a specific level of organization of an infectious disease system. Therefore, our replication-transmission relativity theory for infectious disease systems is about multiscale phenomena ranging from the cellular level to macroecosystem level. This is not new in the application of the relativity principle. Relativistic quantum theory has had remarkable success in dealing with phenomena ranging from the level of the atomic nucleus to the level of the tertiary structure of organic molecules^24^. We anticipate that the replication-transmission relativity theory will remain firmly established as the fundamental theory on which multiscale modelling of infectious disease systems is based on from the cell level to the macroecosystem. Therefore, with a theory in place, we expect that multiscale modelling of infectious disease systems will evolve and expand in scope.
